# The use of insect cell line Sf21 for ecotoxicity testing

**DOI:** 10.1007/s10646-024-02781-9

**Published:** 2024-07-12

**Authors:** Trevor Grigg, Richard D. Handy, Richard A. Billington

**Affiliations:** School of Biological and Marine Sciences, University of Plymouth, Drake Circus, Plymouth, PL4 8AA UK

**Keywords:** Copper sulphate, Propiconazole, Cytotoxicity assay, Insect cells, Agrochemical testing, Sf21 cells

## Abstract

Insect cell lines are finding utility in many areas of biology, but their application as an in vitro tool for ecotoxicity testing has been given less attention. Our study aimed to demonstrate the utility and sensitivity of Sf21 cells to commonly used fungicides: Propiconazole and CuSO_4_, as well as dimethyl sulphoxide (DMSO) an industrial solvent. Sf21 cells were readily cultured from frozen stocks in 3-4 days and showed utility as an invertebrate in vitro acute toxicity test. The data showed the threshold levels of cell survivability against propiconazole and CuSO_4_. The EC_50_ values were 135.1 μM and 3.31 mM respectively. The LOAEL (lowest observed adverse effect level) was ≈ 1 μM for propiconazole and ≈ 10 μM for CuSO_4_. Culturing of Sf21 cells in media containing the solvent DMSO showed that 0.5% DMSO concentration did not effect cell viability. Sf21 cells are sensitive and useful as a robust ecologically relevant screening tool for acute toxicity testing.

## Introduction

There is an imperative in regulatory toxicity testing to use alternatives to vertebrate animal testing. Such ethical concerns are now also extending to invertebrate species (Drinkwater et al. 2019). Currently, insects are used for in vivo toxicity, especially on agrochemicals (Zhou et al. 2019). However, the extensive use of insecticides has resulted in the development of resistance in many important agricultural pests. Notably, the fall armyworm a major pest of maize crops and cotton is resistant to 29 different insecticides (Gutiérrez-Moreno et al. 2019). Insect cell lines show great promise as an alternative to in vivo toxicology testing systems (Cc et al. 2021, He et al. 2023). Vaughn et al. (1977) established the Sf21 cell line, also called IPLB-SF21-AE, derived from the pupal ovary tissue of the fall army worm *Spodoptera frugiperda*. The Sf21 cell line potentially offers utility for the rapid screening of novel biocides against this pest.

Propiconazole is a commonly used azole fungicide that has been commercially available since the 1960s. However, azole fungicides are persistent in the environment. For example, propiconazole in sandy loam soil has a half-life of about 315 days (Kim et al. 2015). Furthermore, there are concerns of ecotoxicity to non-target organisms when used as a fungicide on crops (Hof 2001). Propiconazole disrupts the biosynthesis of ergosterol, the dominant lipid constituent of fungal cell walls via inhibition of the cytochrome P450 system (Ktiller and Scheinpflug 1987). Unfortunately, inhibition of the P450 system in non-target animal and beneficial insects is also a concern. For example, propiconazole was found to have the strongest synergistic effect on honey bee mortality when co-applied with a pyrethroid insecticide (Pilling and Jepson 1993). Furthermore, mammalian hepatocytes are predominantly used for cytotoxicity testing of propiconazole (Chen et al. 2008; Satapute and Kaliwal 2015) and screening with an insect cell line would be more ecologically relevant.

Copper is an essential trace element with numerous biological functions, but may also be toxic in excess with modes of action as an oxidising metal and an ion transport inhibitor (Handy 2003). Copper sulphate is used as a biocide and in crop protection. It is used in trade mixtures against grapevine downy mildew, in fruit orchards and potato farming. Due to copper bioaccumulation in the soil from widespread fungicidal use, and other sources (e.g., copper mining) there are concerns about its effects on non-target organisms such as earthworms (Tatsi et al. 2018) and wildlife in general. There are some reports on Cu toxicity to cell lines from aquatic invertebrates (Anjos et al. 2014; Quinn et al. 2009), but few on insect cells (Braeckman 2011; Raes et al. 2000).

The aim of the present study was to demonstrate the utility of Sf21 cells as a model for screening agrochemicals and here we demonstrate acute toxicity assays on propiconazole and copper sulphate as example chemicals with different modes of action. Reproductive tissue is a target for both chemicals. Copper can be a neuro-endocrine disruptor (Handy 2003) and propiconazole can harm cell membranes (Chen et al. 2008). During the cryopreservation of many cell types the storage medium frequently contains dimethylsulphoxide (DMSO), to prevent ice crystal formation inside the cells. It may also be commonly used as a solvent control in ecotoxicity tests, but its toxicity to Sf21 insect cell lines is not clear. Therefore, an additional aim was to determine how Sf21 cell viability was affected by exposure to varying DMSO concentrations, as a wider part of demonstrating the utility of the cells for toxicity testing.

## Methods

### Sf21 cell culture

**Fig. 1:**
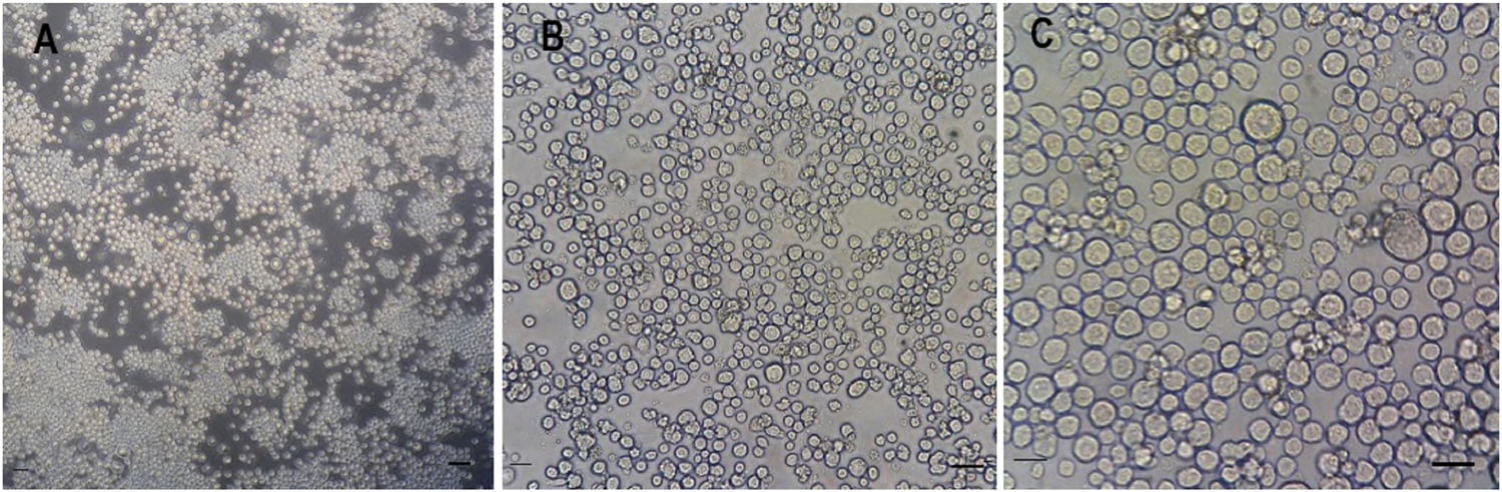
Phase contrast microscopy images of Sf21 cells grown as an adherent monolayer. Magnification (**A**) ×100, (**B**) ×200, (**C**) ×400. The cells were plated at a seeding density 5 × 10^4^ viable cells/cm^2^ in a T-75 flask. Images were obtained 10 days after seeding when the culture had reached ≈ 70% confluency. Bar (**A**) 50 μm, (**B**, **C**) 20 μm

Sf21 cells (Fisher Scientific, UK) derived from pupal ovary tissue were maintained at 28 °C in serum free SF900 II cell culture media at a pH of 6.2 (Fisher Scientific, UK) supplemented with antibiotic 1% penicillin/streptomycin. Cells were seeded at 6.7 × 10^4^ cells/cm^2^ in standard 75 cm^2^ (T-75) culture flasks (Greiner Bio One) to ≈70% confluence. A subculture was performed every 4-5 days to obtain enough cells for the experiments with the passage number recorded. Images of an established cell culture are shown in Fig. 1.

#### Freezing and thawing of Sf21 cells

The toxicology testing was done on thawed cells grown to ≈70% confluence from previous cryopreserved stocks, as might normally be the case in a commercial ecotoxicity testing laboratory. Cell viability prior to seeding in new T75 flasks and prior to cryopreservation was checked for ≥90% viability by the Trypan blue test. Briefly, an aliquot of cell suspension was diluted 1:4 with 0.4% Trypan blue solution (Sigma, UK), and allowed to incubate for 2 min. Cells were counted manually on a haemocytometer. Viable cells look clear and non viable cells appear blue and the percentage of viable cells calculated. The cryopreservation medium consisted of sterile filtered SF900 II conditioned medium (46.25% v/v), SF900 II fresh medium (46.25% v/v) without antibiotic and DMSO (7.5% v/v) (ultra pure, Sigma,UK). For frozen storage 5 × 10^6^ cells in 1 ml aliquots of cryopreservation medium were stored in 2 ml cryogenic vials (Elkay Products, UK) followed by overnight gradual freezing using a Mr Frosty Nalgene Cryo 1 °C freezing container to −80 °C. Vials were subsequently stored in the vapour phase of liquid nitrogen. For thawing and resuscitating cells for experiments. 5 × 10^6^ cells in a cryovial were thawed in air on the bench and quickly transferred with a sterile Pasteur pipette into a T-25 flask containing 10 ml of SF900 II cell culture medium without antibiotic. Cells were kept at 28 °C for 1 h to attach, before discarding the DMSO containing medium and replacing with 5 ml medium containing antibiotic (as above). Cells were placed in an incubator at 28 °C.

#### Characterising the cells for ecotoxicity studies

For in vitro toxicology where cell growth or survival is used as an endpoint, it is important to understand the normal growth and doubling-time of the cultures, so that the health of the cells used in any subsequent toxicity test can be confirmed. To determine the kinetic growth curve of Sf21 cells in continuous culture, cells were maintained for 13 days at 28 °C in SF900 II medium with 1% penicillin/streptomycin antibiotic. Cells were seeded at 6.7 × 10^4^ cells/cm^2^ in a T-75 flask using a one time passaged subculture from cryopreserved stock. Attached cells were counted on seven separate days using an eyepiece graticule grid at 100× magnification on a phase contrast microscope. The total number of cells in 40 graticule squares of a total area of 4.2436 × 10^−1^ mm^2^ was recorded. At day 13, cells were 100% confluent and passaged in a 1:4 split. Doubling times were calculated from the slope of the linear portion of a semi-log plot of cell quantity vs time. A plot using the base_2_ logarithm of cell quantity and the reciprocal of this slope represents the cell doubling time.

### Cytotoxicity experiments

#### Exposure of Sf21 cells to propiconazole or CuSO_4_

For each assay, 9 × 10^4^ cells/well were seeded in a 24 well plate (Costar, Corning Inc, USA) in 500 μl of SF900 II media supplemented with 1% penicillin/streptomycin and cultured at 28 °C until ≈ 70% confluent. Neat propiconazole (CAS No. 60207-90-1), which has a treacle like consistency (45642, 98.45% pure, Sigma, UK) was initially prepared to a 134 mM concentration dissolved in 100% DMSO and further diluted with medium to achieve desired concentrations. After 72 h of incubation to confluence in normal media, the medium was discarded, and replaced with same SF900 II medium containing eight differing concentrations of propiconazole (10 μM to 10 mM), to a final volume of 1000 μl per well.

Similarly, for CuSO_4_ (CAS No. 7758-99-8, purity minimum 98%, Sigma-Aldrich, UK) exposure, the eight different test concentration treatments were (10 μM to 30 mM) to a final volume of 1000 μl per well. CuSO_4_ and propiconazole treatments were maintained at 28 °C in the dark for 24 h and 48 h respectively. A longer duration of exposure for propiconazole was used as preliminary trials showed toxicity effects were slower to take effect. Controls for both treatments were medium only. Each assay consisted of 3 replicates with cell viability counted at the end of exposure.

### DMSO toxicity test

DMSO is potentially harmful to the cell membrane and may cause increased membrane permeability. A 7.5% of DMSO concentration was present in the highest (10 mM) propiconazole concentration treatment. The final DMSO concentration did not exceed 1% in the lower dilutions of propiconazole treatments (10 μM to 1 mM). It was necessary to determine how cell viability is affected by varying DMSO concentrations for a 48 h period for a range of percentage concentrations as shown in Fig. 4. No DMSO solvent was used in the CuSO_4_ treatments as it is readily soluble in water whereas propiconazole is not. Briefly, 24 well culture plates (Thermo Fisher, UK) were seeded with 1 × 10^5^ cells/well in SF-900 II culture media (including 1% penicillin/streptomycin antibiotic) and incubated at 28 °C for 5–6 days until ≈ 70% confluent. Media was then removed and replaced with fresh media having DMSO concentrations ranging 0 to 10% (v/v) (Fig. 4) for a 48 h period of exposure period at 28 °C and kept in the dark. Cell viability was subsequently assessed by the MTT assay after the 48 h period.

### MTT assay to measure cell viability after exposure to toxins

Cell vitality was evaluated by colorimetrically measuring the reduction of yellow MTT (3-(4,5-dimethylthiazol-2-yl)-2,5-diphenyl-2H-tetrazolium bromide) to purple formazan. MTT enters the mitochondria of viable cells and is reduced by dehydrogenase enzymes to insoluble formazan crystals which can be solubilised and spectrophotometrically quantified at 560 nm. The number of viable cells is proportional to purple formazan absorbance (Twentyman and Luscombe 1987). After the 24 h and 48 h exposure periods, as appropriate, the experiment was halted and the media was carefully removed so as not to dislodge cells and replaced with 450 μl control media and 50 μl of MTT (5 mg MTT in phosphate buffered saline (PBS) with Ca^2+^/Mg^2+^), to give a concentration of 0.5 mg ml^−1^ MTT, and incubated for 3 h at 28 °C in the dark. Formazan crystals were solubilised by adding 500 μl DMSO and gently shaken for 30 min in the dark. The absorbance (A) was read at 560 nm in a micro-plate reader (FLUOstar Omega 415, BMG Labtech, UK) and the data processing software was Soft max Pro 5.4. Cell viability is indirectly proportional to the A_560_ data.

### Statistics

Curves for propiconazole and CuSO_4_ dose response experiments were drawn with SPSS version 24.0 and the EC_50_ values calculated by probit analysis. Differences were assessed with the Kruskal–Wallis non parametric test with a post hoc Dunn’s Multiple Comparison test (Bonferonni method). Results with *p* < 0.05 were deemed significant. Statistical analysis of DMSO concentration comparisons were performed using R (R Core Team 2013).

## Results

### Growth curve for Sf21 cells

**Fig. 2:**
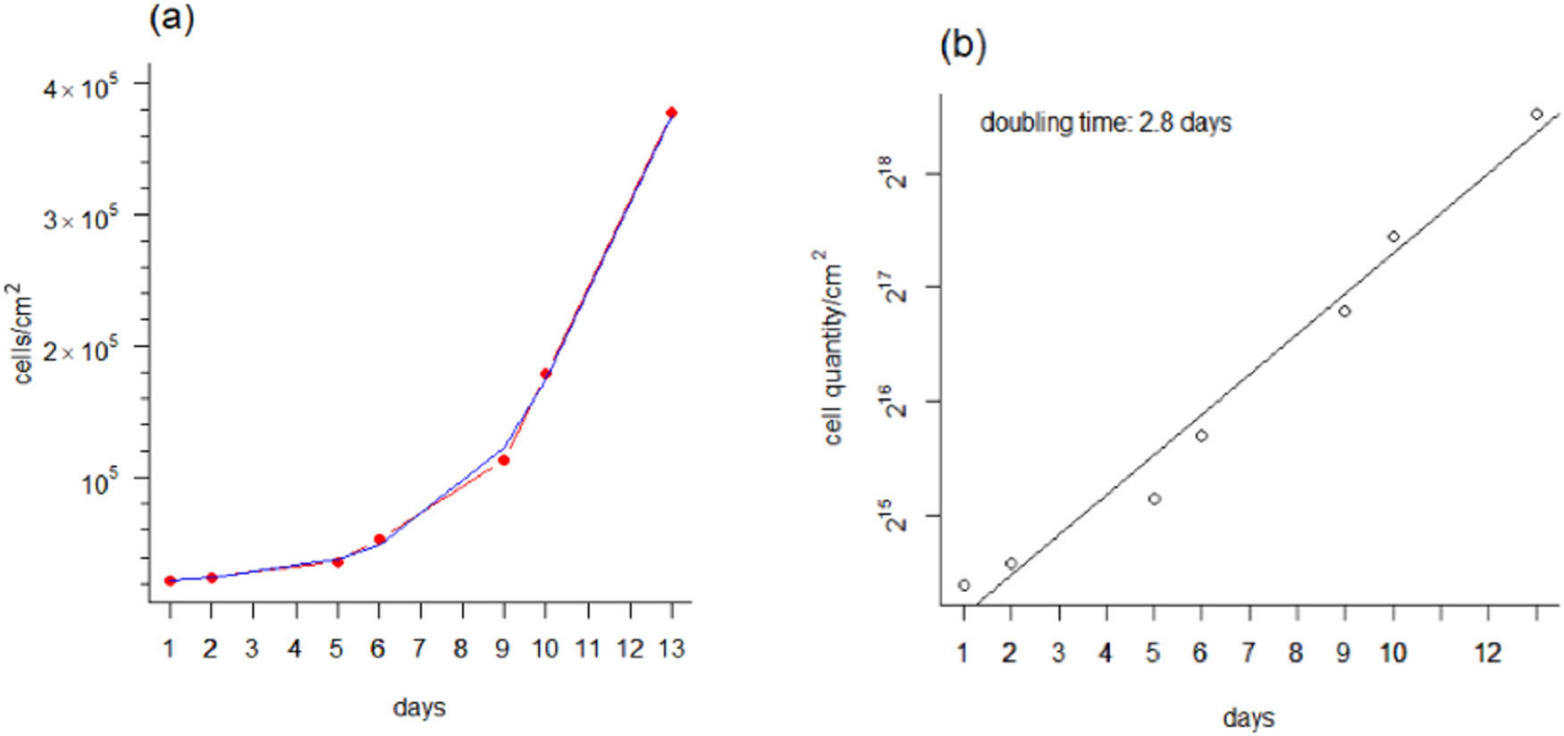
Sf21 cells were cultured from a one time passaged culture (from a previous cryopreserved stock) for 13 days at 28 °C in SF900 II media supplemented with 1% pen/strep in a T-75 flask seeded at 6.7 × 10^4^ cells/cm^2^. **a** Exponential growth curve with cell counts on 7 separate days. Note the initial lag phase as cells recover before continuing to grow exponentially. **b** semi-log_2_ plot of cell quantity increase over time. Cell doubling time was 2.8 days derived as the reciprocal of the growth rate constant 0.3526 from the depicted straight line of equation y = 0.3526x + 13.7648 *n* = 1

Sf21 cells are characteristically lymphoblast like in shape, and of an irregular spherical size of 15–25 μm diameter. Cell morphology showed no signs of membrane blebs or cell shrinkage which are frequent indicators of apoptosis or cellular stress (Fig. 1). Figure 2 shows the cells multiplied in a typical exponential growth pattern. There was an initial lag phase of 4 to 5 days as the cells recovered their capacity for cell division following passaging. This was followed by a period of exponential growth, indicating that cells were not limited by the media. The growth pattern showed no indication of plateauing and entering a final stationary phase even though the monolayer of attached cells had become almost 100% confluent by day 13. The initial seeding density of 6.7 × 10^4^ cells/cm^2^ in a T-75 flask is equivalent to 5 × 10^6^ cells/flask, which is the upper limit of the seeding density range (3.75–5 × 10^6^ cells/flask) for viable cells as recommended by the supplier. Cells rapidly attached (within 30 min), and after a few days entered a rapidly dividing log phase growth period with a doubling time of 2.8 days or 67 h (Fig. 2b).

### Cytotoxicity of propiconazole and CuSO_4_ in Sf21 cells

**Fig. 3:**
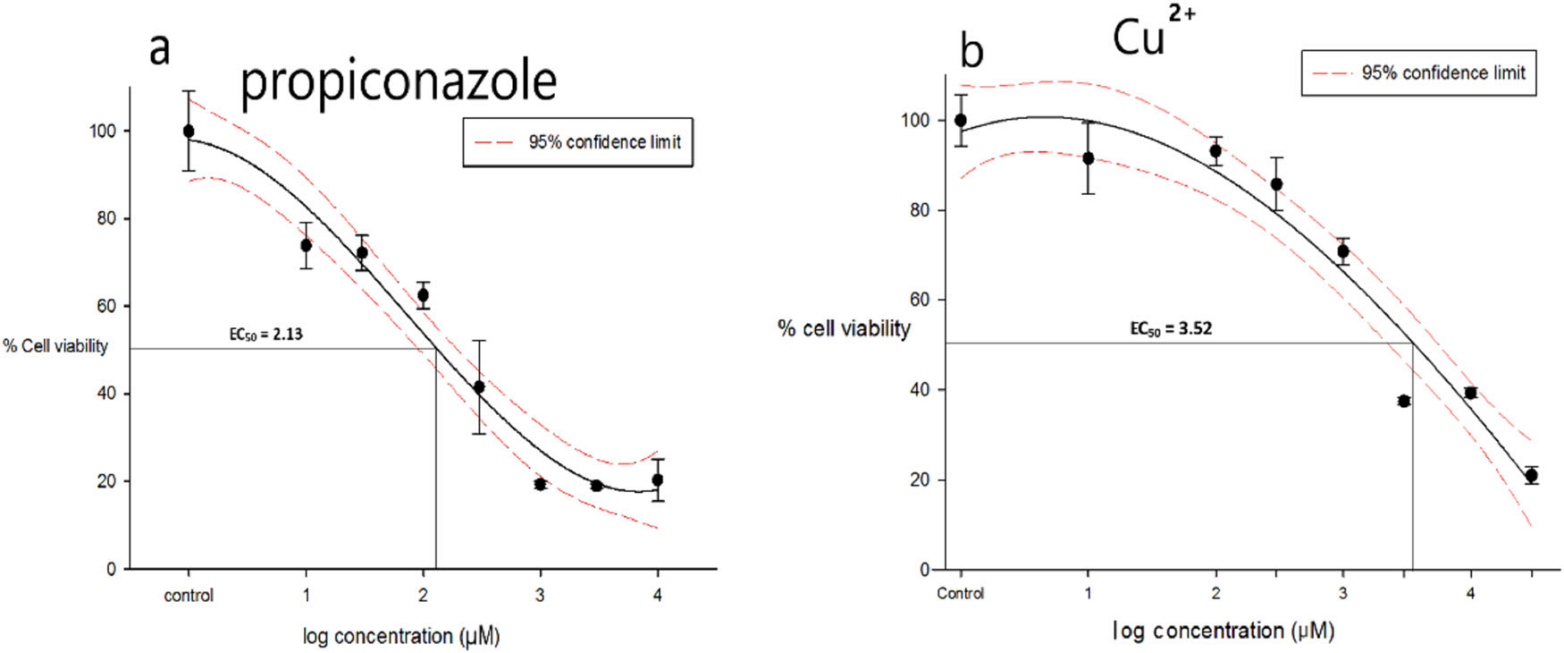
Tolerance of Sf21 cells to various propiconazole and CuSO^4^ concentrations. EC_50_ shown as a base_10_ logarithm value represents the toxin concentration (μM) at which 50% of the cell population can remain viable. The percentage of viable cells is based on the MTT cell viability assay. **a** Concentration dependent decline in Sf21 cells viability after propiconazole treatment for 48 h. (EC_50_ = 2.13 ± 0.14, antilog = 135.10 μM). **b** Concentration dependent decline in Sf21 cells viability after CuSO_4_ treatment for 24 h. (EC_50_ = 3.52 ± 0.15, antilog = 3.31 mM) All statistical analyses were performed with SPSS version 24.0. EC_50_ values were calculated using probit analysis. Shown are averages with standard deviations (error bars). *n* = 3

The dose-response plot for cells exposed to propiconazole for 48 h followed the expected sigmoid survivorship curve (Fig. 3a). The lowest observed adverse effect level (LOAEL) was ≈ 1 μM as extrapolated from the plot (Fig. 3a). As the dose increases to ≥10 μM, the lethality increased exponentially until ≈ 1 mM when the effect ceased to get bigger. A 100% mortality was not obtained. At the highest concentration of 10 mM ≈ 20% of cells were still recorded as being viable. The EC_50_ = 135.1 μM (95% C.I. = 89.1–208.9 μM).

The dose-response curve for cells exposed to CuSO_4_ for 48 h does not fit a sigmoid curve but is an inverted half U shape (Fig. 3b). The LOAEL is seen at the experiment’s lowest concentration of 10 μM and cell viability declines exponentially (linearly on a log scale) at concentrations above 100 μM until approximate 20% viability at the highest concentration of 30 mM. The EC_50_ = 3.31 mM (95% C.I. = 2.09–5.89 mM).

**Fig. 4:**
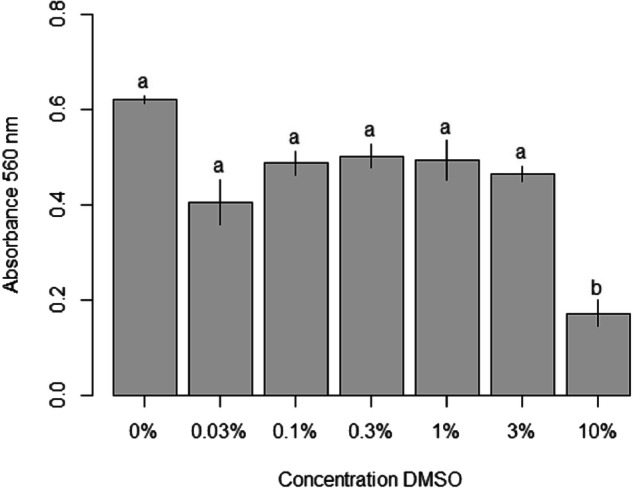
Effect of DMSO concentration on Sf21 cell viability after 48 h exposure as determined by the MTT assay. Data are expressed as mean ± SE. A Kruskal–Wallis test confirmed that the treatments were dissimilar from one another with different lowercase letters indicating significant differences between treatments (*p* < 0.05). A further Dunn’s test determined the 10% treatment to be significantly different. Bars represent SEM, *n* = 3

The effect of DMSO (0.0–10.0%) on Sf21 cell viability as measured by the MTT assay is shown Fig. 4. As the histogram bars of 0.3% and 1% compared to control of 0.0% are not significantly different. So it can be inferred that DMSO at 0.5% concentration in the cell culture media has a non significant effect on Sf21 cell viability. However, at a 10% concentration a significant (*p* = 0.004) reduction in cell viability is observed as shown by Fig. 4.

## Discussion

### The growth and passaging of Sf21 cells

Microscopic observation of cell morphology consistently showed healthy cells of the expected form and structure (Fig. 1), and consistent with previous reports of cell morphology (ThermoFisher 2023). The growth of the cells was also good with a population doubling time of 67 hours (Fig. 2), that was consistently obtained even after the cell populations had been frequently passaged or recovered from cryopreservation. From the viewpoint of ease of culturing for routine toxicity testing, the cells reached confluence in serum-free media within a few days, and without the need for gassing the cells with air/CO_2_ mixtures. Typically, the cells would be ready for acute toxicity testing (lethality studies) within 3-4 days after thawing. This is comparable to other cell lines that are used when confluent after a few days or a week (e.g., rainbow trout gill cells, (OECD 2021)). In addition, because of the exponential invertebrate cell biomass growth under nutrient sufficient conditions (Fig. 2), the Sf21 cells used here might also find utility using growth rate as an alternative endpoint. The lag phase is a few days, but the cells could be used for a growth inhibition assay after day 6 (Fig. 2a). Acute toxicity tests based on growth inhibition are well known (e.g., OECD 2011) on algal growth inhibition, and also the minimum inhibitory concentration (MIC) assay for microbes (Vassallo et al. 2018). A similar approach could be developed for insect cell culture, and as yet, there are no approved in vitro tests with insect cell lines at the Organisation for Economic Cooperation and Development (OECD), the International Organisation for Standardisation (ISO), or similar bodies.

### Propiconazole and CuSO_4_ toxicity

Sf21 cells are sensitive to propiconazole with a 48 h EC_50_ of 135 μM and LOAEL of ≈1 μM (Fig. 3a). Survival exponentially declines in the manner of a traditional sigmoid response curve as dose is increased. To our knowledge, this is the first study of propiconazole toxicity in an invertebrate cell line, but the findings here are broadly comparable to studies with mammalian cells: human HepG2 cells with a 24 h LC_50_ of 120 μM (Satapute and Kaliwal 2015) and 148 μM (Chen et al. 2008) and murine NIH/3T3 cells with a 24 h LC_50_ of 214 μM (Li et al. 2013). Our new assay might be an easier, less expensive method for the initial acute toxicity screening of propiconazole levels than using mammalian cells. It would be helpful for tiered approaches to hazard assessment if the cell culture screening tool could be predictive of in vivo testing. In bees, the 48 h acute oral toxicity of propiconazole is around 40 μg per bee (approximately 1 μM per bee) (Ladurner et al. 2005). In acute aquatic toxicity tests, the algal growth inhibition test reported a 72 h IC_50_ of 1.14 μM for propiconazole, and an LC of 26.3 μM on the *Daphnia* test respectively (Ochoa-Acuña et al. 2009). These are all at least an order of magnitude more sensitive in vivo than the in vitro cell culture methods above, but as a screening tool the cell culture of Sf21 cells could be used to raise concern for toxicity as first tier of integrated testing strategy. Interestingly, Sgolastra et al. (2018) reported decreased fecundity of the solitary bee *Osmia bicornis* using a dose equivalent to the recommended field rate of 0.25 l/ha of a commercial propiconazole formulation; this equated to 183 μM of propiconazole which is close to the EC_50_ of 135.1 μM obtained here with Sf21 cells.

Sf21 cells exposed to CuSO_4_ had a 24 h EC_50_ of 3.31 mM (Fig. 3b) and showed concentration-dependence as expected. There was no hormetic effect observed in this study, but it is possible that much lower doses of Cu could cause hormesis since it is a nutritionally required metal. There are only a few previous studies on Cu toxicity using insect cell cultures. Raes et al. (2000) and Braeckman (2011) found CuSO_4_ was toxic to C6/36 cells from the mosquito (*Aedes albopictus*) with 24 h LC_50_ around 1 mM, and similar to this study which is of a similar magnitude to our EC_50_ of 3.3 mM. There are no studies on Sf21 cells with Cu but murine fibroblasts (NIH-3T3 cells) revealed a 24 h EC_50_ of 0.29 mM (Rakers et al. 2014). Copper toxicity to rainbow trout skin cells (OMYsd1x) had a 24 h EC_50_ of 1.65 mM (Rakers et al. 2014). So the study here with Sf21 cells is broadly similar to other cell cultures on vertebrate animals. Only the rainbow trout gill cell line (RTgill-W1) is more sensitive with an EC_50_ around 0.63 μM of free Cu^2+^ after 2.5 h exposure to CuSO_4_ (Bopp et al. 2008). However, this might be expected given the sensitivity of fish gills to metals. A further aspect is whether the Sf21 toxicity test could be used for ranking chemicals of concern. The cells showed sensitivity to both propiconazole and CuSO_4_. The EC_50_ values suggest that propiconazole is more toxic than CuSO_4_ to insect cells and the latter might be safer to non-target invertebrates as a fungicide on crops. The acute test with Sf21 cells potentially offers a rapid screening tool. Chronic tests have not been done with this insect cell line, but the LC_10_ from the acute toxicity plot could be used to inform on likely chronic toxicity. Regarding extrapolation of cell line results to in vivo, more data is needed on insect cells, but the EC_50_ here is close to the application rate of propiconazole and its effect on bees (above). In addition, more studies on exposure of Sf21 cells in comparison to effects on insect reproductive tissue in vivo are needed for any cell to in vivo extrapolation of the hazards.

As regards to the thresholds (LOAEL) at which an effect begins to occur, CuSO_4_ has the higher threshold concentration at about 10 μM compared to around 1 μM of propiconazole (Fig. 3a). The dose-response curves are tight fitting with predominantly low standard deviation variation for most data points which lie within or on the 95% confidence limits, and ranking chemicals using the confidence interval (CI) overlap method may also be possible (Payton et al. 2003). Comparing the CIs from propiconazole and CuSO_4_ (Fig. 3a, b) there is no overlap of values and one could conclude that propiconazole and CuSO_4_ have different toxicities. Another method to compare the lethality is the Equivalency Factor (EF) that can be applied when dose-response slopes are approximately equal (Mayo et al. 2015). It is the ratio of EC_50_ values. In this study the EF is = EC_50_ Cu^2+^ ÷ EC_50_ PPZ ≈ 25 indicating propiconazole is 25 times more hazardous.

### Effect of DMSO

DMSO is routinely used as a solvent to disperse poorly soluble organic chemicals for toxicity tests (Modrzyński et al. 2019) and typically at concentrations around a few percent. DMSO concentrations of ≤3% had no effect on Sf21 cell viability after 48 h of exposure, although 10% DMSO had an adverse effect (Fig. 4). Arguably, the 1% DMSO concentration or less in the propiconazole experiments was not a hazard to Sf21 cell viability. There appears to be no other reports of the cytotoxicity of DMSO on Sf21 or related Sf9 cells. In eucaryotic cells the maximum dose should be 0.5% DMSO for routine cell culture (Pal et al. 2012), although in other reports a noticeable decrease in stem cell viability occurred at DMSO concentrations of 3 and 5% (Lee and Park 2017). There was some cytotoxicity at 1% with fibroblasts (Chen and Thibeault 2013). For plant cells, 1.4% DMSO concentration in the culture media did not affect cell growth (Schmidt et al. 1989). There are concerns that DMSO may influence the results of fish early life stage toxicity tests (Kais et al. 2013), but DMSO used as a solvent in whole effluent tests at least had negligible effects on rainbow trout gill cells (Dayeh et al. 2002). Overall, Sf21 cells are quite resilient to DMSO and the doses typically used in ecotoxicity tests are unlikely to effect them.

## Conclusion

Attached Sf21 cells cultured for ten days in the presence of antibiotic routinely achieve 70% confluent growth and a density of ≈ 2 × 10^5^ cells/cm^2^. The stable growth characteristics, microscopic observations, ease of culture and tolerance to the two xenobiotics studied make Sf21 cells a suitable insect cell model for future toxicity testing experiments. In addition, we show characteristic growth curves of Sf21 cultures and its usefulness as a robust experimental model for assays of invertebrate cell death from fungicides propiconazole and copper sulphate as well as the solvent DMSO. The Sf21 cells offer utility for ecotoxicity testing and would be relevant to agrochemical safety and the screening of fungicides and other biocides used in agriculture. Useful also for determining the representational sensitivity to those pesticides, for which *S. frugiperda* larvae and adults have developed resistance. Further work is needed for standardisation and to move towards an agreed regulatory test method for insect cell cultures.
